# Vesicle fusion and release in neurons under dynamic mechanical equilibrium

**DOI:** 10.1016/j.isci.2024.109793

**Published:** 2024-04-19

**Authors:** Wenhao Liu, Tianyu Gao, Na Li, Shuai Shao, Bo Liu

**Affiliations:** 1 Cancer Hospital of Dalian University of Technology, Shenyang 110042, China; 2 Faculty of Medicine, Liaoning Key Lab of Integrated Circuit and Biomedical Electronic System, Dalian University of Technology, Dalian 116024, China

**Keywords:** Neuroscience, Biophysics

## Abstract

Vesicular fusion plays a pivotal role in cellular processes, involving stages like vesicle trafficking, fusion pore formation, content release, and membrane integration or separation. This dynamic process is regulated by a complex interplay of protein assemblies, osmotic forces, and membrane tension, which together maintain a mechanical equilibrium within the cell. Changes in cellular mechanics or external pressures prompt adjustments in this equilibrium, highlighting the system’s adaptability. This review delves into the synergy between intracellular proteins, structural components, and external forces in facilitating vesicular fusion and release. It also explores how cells respond to mechanical stress, maintaining equilibrium and offering insights into vesicle fusion mechanisms and the development of neurological disorders.

## Graphical abstract

**Figure:**
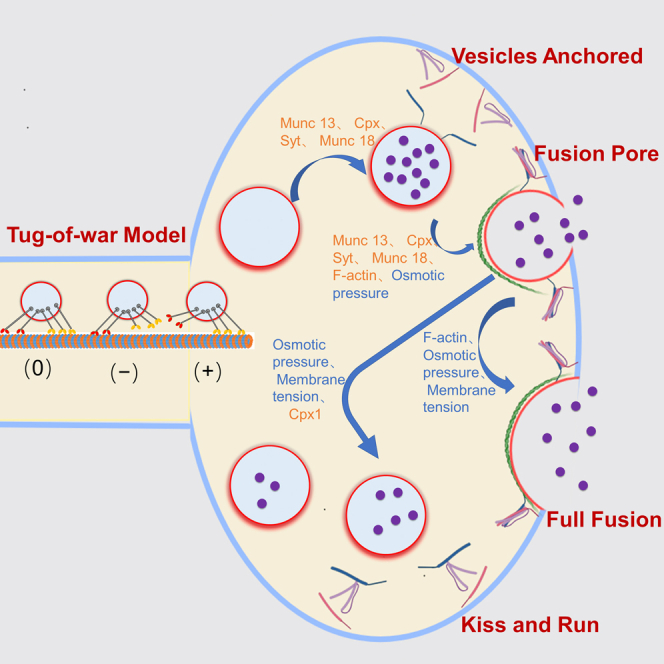

## Introduction

Vesicle fusion and release of vesicle contents, such as hormones and neurotransmitters, play critical roles in many essential biological processes in animals, including stress response, emotional changes, synaptic transmission, neural network activity, and immune response.^1^^,^^2^ Existing studies have shown that the process of vesicle fusion and release is in a dynamic mechanical equilibrium under the combined effects of multiple factors, such as cell structure, proteins, and intracellular and extracellular stresses.^3^^,^^4^^,^^5^ The cell skeleton can provide a track for vesicle transport, while actin, myosin, and the soluble N-ethyl-maleimide-sensitive factor attachment protein receptor (SNARE) complex provide the force for fusion and release during the process. Vesicles arrive at the plasma membrane, and an Ω-shaped intermediate structure (called Ω-profile) is formed through membrane tension for the release of contents. The opening of Ω-profile is the fusion pore, which closes in the “kiss and run” mode or merges itself into the plasma membrane in the “full fusion” mode under the regulation of the stresses generated by actin, synthesis protein, synaptotagmin, and Munc18-1.^6^^,^^7^ On the other hand, the shape and surface changes of membrane can affect its mechanical properties, impacting on the enlargement or reduction of the fusion pore by forming a negative feedback loop. Increasing evidence suggests that cell-internal mechanical stress factors, such as membrane tension and motor proteins, Cdc42 protein, F-actin, SNARE complex, integrins, play crucial roles in regulating vesicle fusion and release.^8^^,^^9^

The vesicle fusion and release of cells are a process that interacts with the microenvironment. The release of vesicle contents, such as neurotransmitters, hormones, and exosomes, allows cells to communicate with the microenvironment via autocrine and paracrine modes.^10^ The deposition of extracellular matrix proteins and matrix metalloproteinases (MMPs) further enables cells to actively reshape their microenvironment.^11^^,^^12^ In addition to molecular secretion, vesicle fusion also brings the intrinsic components of the plasma membrane, such as lipids and membrane-related proteins.^10^ These components can affect the physiological state of cells, modify the activity and assembly of actin, regulate the dynamics of the actin cytoskeleton, and thus affect the original dynamic equilibrium of vesicle fusion and release. Receptor tyrosine kinases (RTKs) and integrins, for example, recognize extracellular ligands, sense chemical and mechanical factors in the environment, and then transmit signals into the cells, allowing cells to adapt to the environment and achieve a new dynamic mechanical equilibrium.^13^^,^^14^

Neuronal cells typically do not experience tensile stress. However, during incidents such as vehicular accidents or sports injuries, cells inevitably undergo stretching or compression. Furthermore, in associated neurodegenerative diseases, such as hydrocephalus and brain tumors, the accumulation of cerebrospinal fluid can lead to increased intracranial pressure. The abnormal growth of cells may exert pressure on adjacent cells, thereby subjecting neurons to tensile stress. Such tensile stress can affect the aggregation of vesicles, actin, and microtubules in synaptic regions.^15^^,^^16^^,^^17^

When examining the impact of external stress on vesicular fusion and release, shear stress cannot be overlooked. This is attributable not only to the typical interaction of cells with a fluidic environment but also to the fact that the normal growth and movement of secretory cells, and their inevitable interaction with surrounding tissues or other neurons, subject them to shear stress. Shear stress can modulate vesicular fusion and release by regulating ion channels, receptor proteins, and membrane tension associated with vesicular fusion.^18^^,^^19^^,^^20^^,^^21^

Cells perceive various types of external stress stimuli through different signaling pathways, transform them into changes in intracellular stress, and subsequently break the original dynamic mechanical equilibrium of vesicle fusion and release. The related mechanical structures and proteins inside the cells respond to the stress, guiding the process of vesicle fusion and release to achieve a new dynamic mechanical equilibrium.

This article provides a comprehensive review of the dynamic mechanical equilibrium involved in the key stages of vesicle fusion and release, analyzing how cells respond to changes in stress conditions, adjust the process of vesicle fusion and release, and achieve new dynamic mechanical equilibrium. The dynamic equilibrium model outlined in this article effectively elucidates the operational mechanisms of vesicle fusion and release, providing a reference for studying diseases related to abnormal vesicle fusion and release.

### Stress changes during vesicle fusion and release

Vesicle fusion and release constitutes a complex and precise sequence of events. Precursors of vesicles are subjected to processing and modification within the Golgi apparatus and the endoplasmic reticulum, followed by additional steps including vesicular transport, the formation and closure of fusion pores, and the ultimate release of vesicular contents. Each step is regulated by mechanical factors related proteins, osmotic pressure, and membrane tension to maintain a dynamic mechanical equilibrium, which is essential for normal vesicular fusion and release processes.

#### Vesicular transport

**Figure 1:**
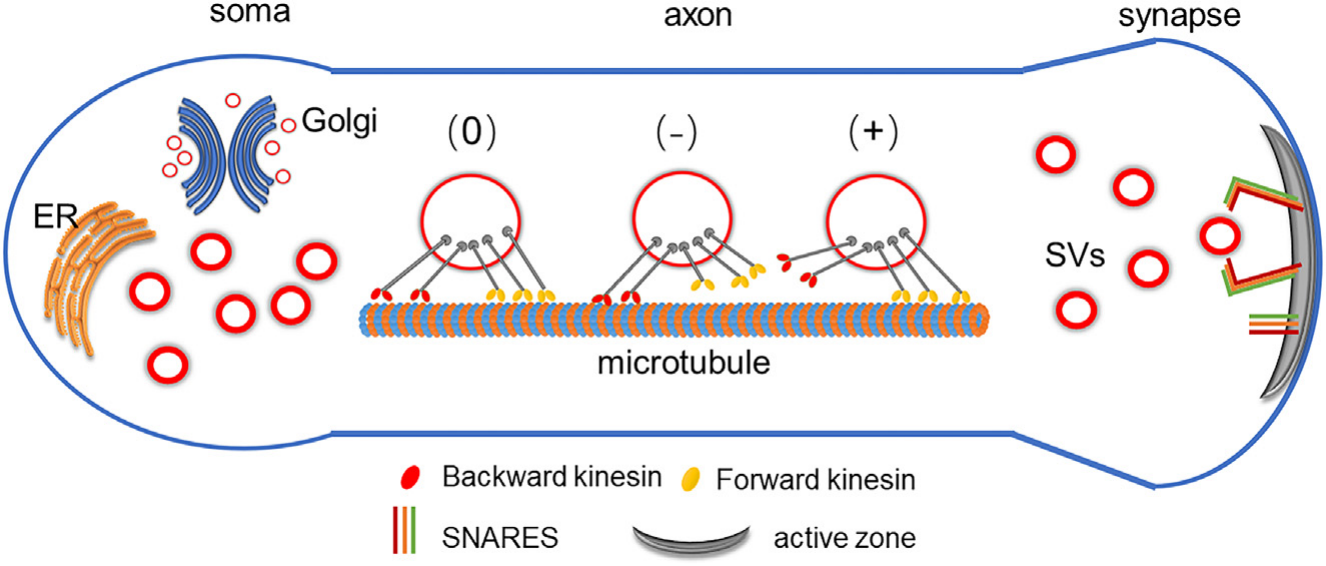
Vesicular Transport Vesicular transport is mediated by motor proteins that drive movement in an anterograde (positive) or retrograde (negative) direction. The potential configurations of the motors bound to microtubules (MT) are denoted as (0), (+), and (−). In the (0) configuration, the motors are in stasis due to mutual obstruction, resulting in no net movement of the cargo. Configurations (+) and (−) correspond to rapid anterograde and retrograde movements of the cargo, respectively.

After vesicular precursors are formed in the cell body and processed by the Golgi apparatus and the endoplasmic reticulum, they are transported to the synaptic region via a specialized axonal transport system made up of microtubules, cytoskeletal tracks, and numerous molecular motors. Multiple motor proteins interact to drive bidirectional vesicular transport.^22^^,^^23^ Müller et al. proposed a “tug-of-war” model to explain the mechanical process of vesicular transport. In the absence of external factors, the transport of vesicles along axons is regulated by the force of opposing-direction motor proteins. Cytoplasmic dynein tends toward unidirectional movement to the microtubule’s negative end, and kinesin motors toward the positive end. Both types of motor proteins possibly connect to the vesicle simultaneously, dynamically regulating the vesicle transit. In the tug-of-war model, individual motors operate autonomously, engaging in mechanical interactions solely through the shared cargo, with the model encapsulating seven potential mechanisms of cargo transport motion. Of these, three states—designated as (0), (+), and (−) in Figure 1—govern the system, corresponding to stasis of the cargo, rapid movement in the forward direction, and rapid reverse motion, respectively. Additional movement states represent combinations of these primary states, such as (−+) and (−0+), indicative of bidirectional rapid transport, both with and without interim pauses. In instances of swift bidirectional motion, it is typically the case that only one type of motor is exerting a pulling force at any given moment, suggesting a coordinated tug-of-war dynamic.^5^ Within certain parameters specific to single motors, such as stall force and microtubule (MT) affinity, distinct motion states were identified. Slight alterations in these parameters can precipitate dramatic shifts in the mode of cargo transport; for instance, transitioning from rapid forward motion to bidirectional movement or complete immobility. This demonstrates the cell’s capacity to finely tune its transport mechanisms by exploiting the transport system’s sensitivity to individual motor characteristics, thereby facilitating regulation in an extraordinarily efficient manner.

The direction and speed of vesicle transit are affected by the number of antagonistic motor proteins attached to each end of the vesicle. A single kinesin (≈5.5 pN) has the capacity to counteract the pull of four to eight dyneins (≈1.1 pN). Throughout the transport process, the number of kinesins may vary from one to four, and the number of surrounding microtubules can also fluctuate between one and three.^24^ This can well explain observations in PC12 cells where vesicles progress at a constant speed for 0.5–2 s then continue to move at another constant speed.^25^ Sahil Nagpal et al. developed a light-driven inhibitor based on the light oxygen voltage 2 trap and release of protein (LOVTRAP) system using the natural autoinhibitory domains of kinesins and dyneins, which can reversibly inactivate endogenous kinesin-1, kinesin-2, kinesin-3, and cytoplasmic dynein. Their research results indicate that kinesin-1 and kinesin control the direction of vesicular movement, while kinesin-2 and kinesin-3 promote long-distance transportation. Inhibiting kinesin-1 and motor proteins enhances transport by reducing competition between relative movements, while inhibiting kinesin-2 and -3 reduces vesicle motility.^26^ The scaffolding protein JIP1 has been posited to act as a switch between anterograde and retrograde transport by mediating interactions between kinesin motors and dynein. The switching mechanism is proposed to be triggered by the phosphorylation of JIP1.^27^ This scaffold-mediated transition may represent a universal mechanism, utilizing distinct scaffolding proteins for varying cargoes.^28^ Notably, while bidirectional molecular motors are commonly found concomitantly on a given cargo, reports of fleeting associations with a singular motor have been documented.^29^

Quantitative characterizations of intracellular and *in vitro* forces suggest a “coordinated model” between kinesins and dyneins to explain the switching of directions in bidirectional vesicle transport. Multiple modulatory proteins are implicated in this dynamic shift of vesicular trafficking. Halo represents the inaugural member of a novel class of transport regulators, acting as a determinant of directional vesicle transport for embryonic lipid droplets. Stall force assays indicate that its ability to switch directionality is attributed to an increased average number of engaged anterograde motors coupled with a decrease in engaged retrograde motors.^30^ During early neuronal development, high expression of BICDR-1 maintains centrosomal positioning of Rab6-positive secretory vesicles by fostering dynein-driven retrograde transport within neurons; in later stages of development, reduced levels of BICDR-1 expression permit anterograde transport of secretory vesicles requisite for axonal growth.^31^ These discoveries suggest BICDR-1 contributes to temporal regulation of secretory transport by modulating transport directionality. Members of the Hook family proteins serve as cargo adapters on early endosomes. In the filamentous fungus Ustilago maydis, the Hook homolog Hok1 interacts with kinesin-3 and dynein. Real-time imaging of motile early endosomes within fungal hyphae suggests a decrease in kinesin-3 motors attached to cargo prior to dynein binding and directional shifts from anterograde to retrograde, implying that Hok1 coordinates bidirectional transport of early endosomes by facilitating the release of kinesin-3 from cargo, thereby supporting dynein-driven retrograde movements in the tug-of-war.^32^ Multiple regulatory proteins, coordinating motor proteins and driving proteins, ensure smooth delivery of vesicles to their fusion sites.

#### Fusion pore

**Figure 2:**
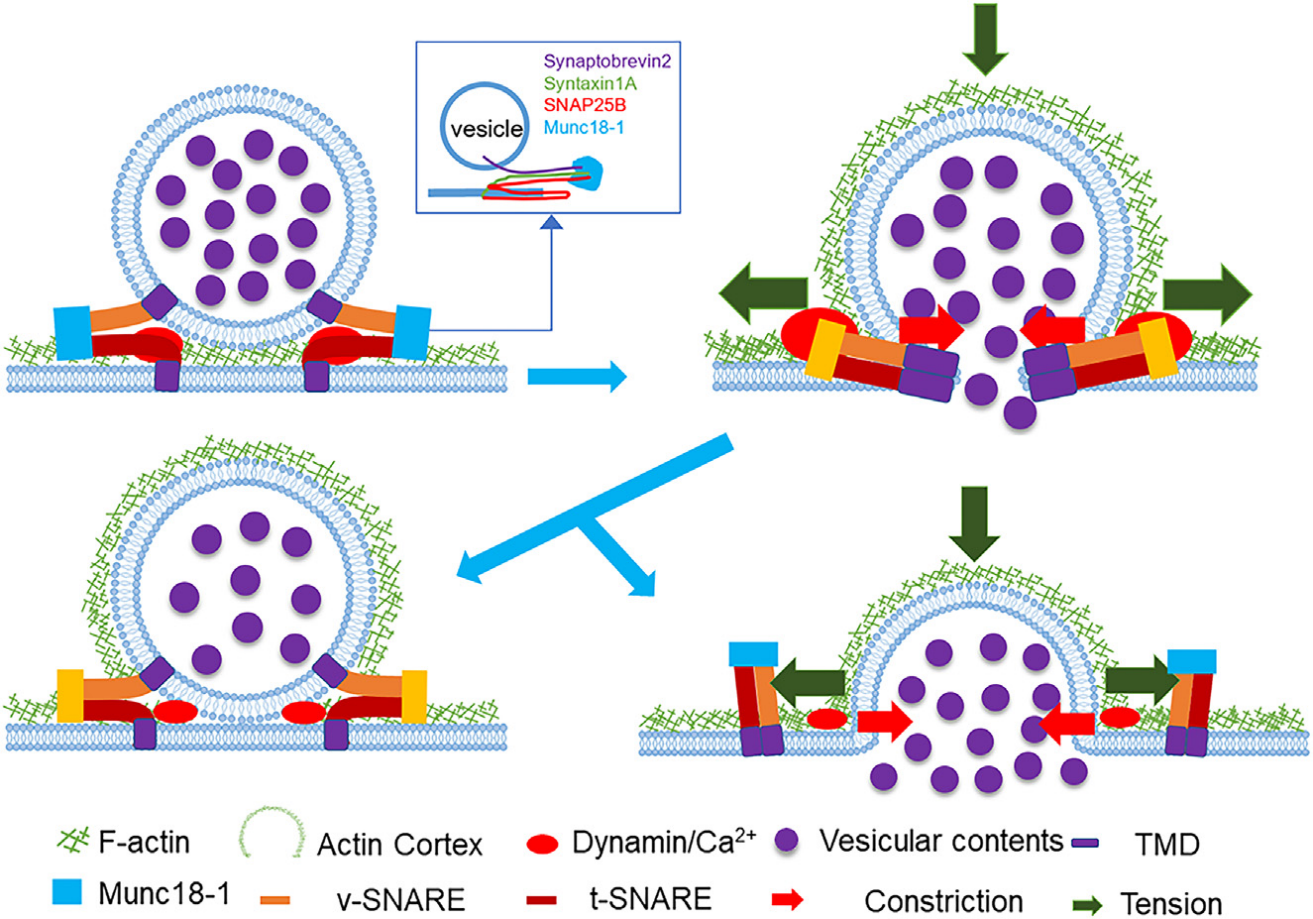
Opening and closure of fusion pore Anchored in the vesicular and presynaptic membranes via their transmembrane domains (TMDs), v-SNAREs and t-SNAREs, in concert with fusion proteins such as Munc13, Cpx1, and Syt1, converge to form SNARE complexes that initiate the opening of a fusion pore through a zippering mechanism. Upon formation of the fusion pore, F-actin-dependent membrane tension contributes to the expansion of the pore diameter, thereby facilitating the release of vesicular contents. The mode of fusion is dictated by the interplay between contractile forces, which tend to close the pore, and expansive forces that promote pore widening.

During vesicle fusion with the presynaptic membrane, a fusion pore is initially formed, through which numerous vesicular contents critical to biological processes—such as neurotransmitters, hormones, enzymes, and cytokines—are released. A diverse array of fusogenic proteins, including synaptotagmin (Syt), complexin (Cpx), Munc13, Munc18, and other tethering factors, work in concert to precisely regulate the vesicular fusion process (Figure 2). Munc18-1 initiates the sequence by capturing Syntaxin 1A (STX1A) and locking it in a closed conformation within a heterodimeric complex, a state that kinetically inhibits the formation of the ternary SNARE complex.^33^^,^^34^ However, this state facilitates the recruitment of Munc18-1 to the active zone fusion sites, where it can act as a template for SNARE complex assembly.^35^ The MUN domain of Munc13 is capable of catalyzing the transition of STX1A from the Munc18-STX1A complex to a ternary *trans*-SNARE complex;^36^ meanwhile, the C2A domain of Munc13 interacts with RIM, thereby modulating vesicle tethering and priming.^37^^,^^38^ By their interactions with the *trans*-SNARE complex, the vesicle membrane, and the presynaptic membrane, Cpx1 and Syt1 lock the vesicle in a pre-fusion state, poised for synaptic release.^39^

In neurons, vesicle-SNARE (v-SNARE) and target-SNARE (t-SNARE) are anchored on synaptic vesicles and the plasma membrane, respectively, through their transmembrane domains (TMDs). Homologous v-SNARE and t-SNARE bind to form SNAREpins via membrane-proximal heptad repeat regions.^40^ These entities form a highly stable alpha-helical bundle structure in a helical intertwining manner, which brings the vesicle and plasma membrane closer together like a zipper and, with the coordinated action of the calcium sensor synaptotagmin-1 (synaptotagmin-1), initiates the opening of the fusion pore.^41^^,^^42^ Given the presence of a hydrophilic layer (“layer 0”) in the center of the SNARE helical bundle, which is nearly 100% conserved, SNAREs can be reclassified into Q-SNAREs and R-SNAREs. A functional SNARE complex, capable of mediating membrane fusion, typically consists of a four-helix bundle formed by three Q-SNAREs and one R-SNARE.^43^ A single SNARE complex can initiate the formation of a fusion pore, but at least three SNARE complexes are required to open a pore large enough to release neurotransmitters.^44^ At the onset of fusion pore formation, the first hydrophobic connection between two adjacent membranes is mediated by the “splaying” of phospholipid acyl chains.^45^^,^^46^ Once the initial hydrophobic connection is established, other hydrophobic acyl chains reorient along the template provided by the splayed chains, moving downwards on a free energy scale toward the fusion stalk, where the proximal leaflets form a highly curved continuous monolayer. The fusion pore opens at the site where the two distal leaflets meet, necessitating the formation of a water pathway through the bilayer, dependent on the reorientation of lipid head groups until the head groups of the proximal and distal membranes meet to form a hydrophilic channel, thereby overcoming the energy barrier to fusion pore formation.^47^^,^^48^^,^^49^ Once the reorientation of the lipid head groups is complete and a water channel lined by head groups is formed, the bilayer becomes stable again, controlling the opening of the fusion pore by minimizing curvature stress and lateral membrane tension.

F-actin-dependent membrane tension is capable of dilating the fusion pore diameter, while Dynamin—a multidomain GTPase—facilitates the constriction/closure of the fusion pore, and calcium influx can also promote pore constriction[13]. The fusion pore diameter, regulated by F-actin-generated membrane tension and calcium/dynamins-mediated actions, can change within a time frame of 26 ms, between 0 and 490 nm (vesicle size: 180–720 nm).^4^^,^^50^

At the same time, syntaxin, as a component on the plasma membrane, is involved in promoting the formation of the fusion pore;^51^ Munc18-1 can enlarge the fusion pore by interacting with syntaxin-1A, Rab3A, and Mint proteins;^52^ sphingosine also enhances the formation of the SNARE complex by activating vesicle associated membrane protein 2(VAMP2) in synaptic and recycling vesicles, further facilitating the pore to enlarge.^53^^,^^54^ Dynamin, synaptotagmin I, and the calcyphosine(CAPS) proteins act to close the fusion pore by enhancing calcium/dynamin-mediated actions and inhibiting SNARE complexes.^55^^,^^56^^,^^57^ Thus, the dynamic equilibrium of the fusion pore is maintained under the collective action of SNARE complexes, actin, syntaxin, synaptotagmin, Munc18-1, dynamin, and CAPS.

Osmotic pressure plays a significant role in regulating the size of fusion pores. Increasing the osmotic pressure of the extracellular solution can shrink cells, thereby lowering the plasma membrane tension and reducing the initial diameter of the fusion pore. Latrunculin A (Lat A), an inhibitor of actin polymerization, reduces the membrane tension in secretory cells. Measuring with stimulated emission depletion (STED) microscopy confirmed the reduction in the initial diameter of the fusion pore. This indicates that actin promotes the dilation of the fusion pore by enhancing the plasma membrane tension.^4^^,^^58^ Influx of calcium ions is associated with an increase in membrane area and a reduction in membrane tension,^59^ suggesting that the calcium/dynamin-mediated constriction of the fusion pore diameter may be achieved through the reduction of membrane tension. Thus, a mechanism may exist in which the enlargement or constriction of the fusion pore diameter depends on changes in membrane tension; and the action of actin, Munc-18-1, dynamin, and synaptotagmin I regulate the membrane tension, thereby controlling the fusion pore. When membrane tension reaches dynamic stability with the involvement of these structures and proteins, the diameter of the fusion pore will also remain at dynamic equilibrium. While factors involved in regulation are perturbed, changes in the fusion pore will follow. For example, the peptide of synaptobrevin-2 (Syb2), which blocks the formation of the ternary SNARE complex,^60^ after introduction of dnSNARE into cells, significantly reduces the diameter of the fusion pore;^61^ using cytochalasin D (Cyto D) to inhibit actin polymerization, it was observed that the rate at which the fusion pore expands was reduced and the duration of the fusion pore’s existence was extended.^62^ These observations can be perfectly explained within the dynamic mechanical equilibrium of membrane tension.

#### Release of contents

Actin can rapidly reorganize itself in response to environmental or internal biological signals, playing a role in several steps of exocytosis. During the pre-fusion stage, actin is involved in regulating the number of vesicles that fuse with the cell membrane;^63^^,^^64^ during transport to the sites of fusion, the actin cytoskeleton provides tracks for vesicle movement^65^ and acts as a scaffold for docking vesicles close to the cell membrane;^66^ through 3D time-lapse imaging in secretory organs, it has been found that actin is abundant at the apical plasma membrane, and actively disassembles at the fusion sites of vesicles,^67^ suggesting actin can serve as a physical barrier to prevent premature fusion of vesicles with the plasma membrane (PM). In Drosophila salivary glands expressing the Lifeact-GFP fluorescent probe, after fusion pore formation, actin reorganizes directionally from the site of PM fusion, covering the fusing secretory vesicles and then moves upward rapidly until the entire vesicle is enveloped.^67^ The actin coat formed plays a pivotal role in mediating the release of vesicular contents.^68^^,^^69^^,^^70^

Under the influence of an osmotic pressure gradient between the intracellular and extracellular solutions, actin provides membrane tension that curls the fused vesicle’s membrane, allowing the release of contents, a phenomenon that has been clearly observed with transmission electron microscopy.^3^^,^^58^^,^^71^^,^^72^ Myosin II regulates this process; inhibiting the activation of myosin II with the chemical inhibitor Y-27632, vesicles still fuse with the apical membrane and form an actin coat around it. If myosin II is not recruited, 60% of the vesicles will fail to constrict. This indicates that the contraction of the actin coat is partially mediated by myosin II.^3^^,^^73^ Rho associated coiled-coil containing protein kinase 1(ROCK1) and myosin light chain kinase 1(MLCK1) complementary regulate the disassembly and cross-linking of actin, thereby providing contractile force for vesicular content release.^3^

The osmotic pressure outside vesicles also plays a role in regulating the release of vesicular contents and interacts antagonistically with the contractile force provided by actin. Gradient osmotic pressure experiments on secretory cells revealed that hyperosmotic solutions could decrease the release of vesicular contents, and even stop the release under sufficiently high osmotic pressures.^74^ The vesicle size also changes under the influence of osmotic pressure; the vesicles in a hyperosmotic environment significantly shrink, and in this process, the concentration of substances inside vesicles remains constant. This indicates that vesicles adjust their volume to maintain a constant concentration of neurotransmitters within, responding to external osmotic pressure changes.^75^

There possibly exists an antagonistic dynamic equilibrium between the osmotic pressure and the actin coat in vesicular content release. The osmotic pressure by compressing the Ω-profile and eliminating its membrane tension generates a tension gradient from the plasma membrane to the Ω-profile, which encourages the fusion of the Ω-profile with the plasma membrane. At the same time, under the compressive force provided by the active contraction of the actin coat,^58^ vesicular contents continue to release. With the continuous reduction of pressure from the squeezing of the actin coat, the osmotic pressure difference between inside and outside the vesicle increases, and the inhibitory force on vesicle content release by osmotic pressure reaches a dynamic equilibrium with the force by the actin coat promoting content release.^58^^,^^76^^,^^77^ At this point, the vesicle no longer releases contents, and the vesicle size stabilizes.

Fusion proteins play an integral role in this process, with the SNARE complex serving as a pivotal driving force during fusion. Complexin-1 (Cpx1), a presynaptic protein, exerts influence over neurotransmitter release by modulating the SNARE complex. In the presence of Ca^2+^, Cpx1 activates synchronous neurotransmitter release through its N-terminal domain and a central α-helical domain that binds to the SNARE complex.^78^^,^^79^ Conversely, in the absence of Ca^2+^, Cpx1 inhibits neurotransmitter release by suppressing SNARE complex assembly.^80^^,^^81^ During the stage of content release, regulatory elements such as the SNARE complex, actin coating, myosin II, Rho-associated kinase 1 (ROCK1), and myosin light chain kinase 1 (MLCK1) facilitate the expulsion of contents. Conversely, the dynamic osmotic pressure, which alters with the diminishing contents, and Cpx1, which dually regulates the SNARE complex and impedes the release of contents, embody a dynamic mechanical equilibrium coupled to the release process.

#### Selection of fusion modes

In past studies on vesicle fusion and release, the fusion modes were divided into two categories: a “full fusion” model for the fusion event after which the vesicle membrane collapses and merges with the plasma membrane,^82^ and a “kiss-and-run” model where the fusion pore opens to release vesicle contents and then closes again.^83^

The vesicle fusion mode is not fixed but dynamically adjusted with different regulatory conditions. Studies using microelectrode amperometry on PC12 cells have shown that kiss-and-run is the predominant mode of fusion.^84^ However, real-time three-dimensional tracking of individual synaptic vesicles in hippocampal neuron terminals has found that vesicles, which are in a long-term stable state, are more inclined to kiss-and-run fusion. After the start of fusion, kiss-and-run fusion events predominantly occur near the center of the synapse, whereas full fusion events are dispersed across the presynaptic membrane area.^85^ The size of the vesicle may also interfere with the choice of fusion mode, with smaller vesicles tending toward full fusion and larger ones more toward kiss-and-run.^86^ Ca^2+^ concentration also regulates the vesicle fusion mode; in chromaffin cells of rats, increasing the extracellular calcium ion concentration shifts the fusion mode from full fusion to kiss-and-run.^50^

Real-time electron microscopy imaging of endocrine cells has shown that during the fusion process, vesicles do not directly collapse onto the plasma membrane and merge with it, rather they shrink constantly in volume along with the release of their contents.^6^^,^^58^ This might reveal another possible vesicle fusion mode—constricted fusion, where the vesicle contracts while maintaining its shape and does not expand the pore. This mode is thought to be mediated by F-actin-dependent plasma membrane tension.^58^ The mechanical regulation mechanism of constricted fusion can match the previously described content release processes, that is, the expansile osmotic pressure maintained by the cell compresses the Ω-profile and eliminates its membrane tension, generating a tension gradient (from PM to Ω-profile), rolling the Ω-profile membrane into PM. When the compressive force equals the cell’s expansile osmotic pressure, the size of the fused vesicle reaches a dynamic equilibrium. After inhibiting actin polymerization with Cyto-D/latrunculin-A (Lat-A), it was observed that vesicle fusion disappeared.^58^ This phenomenon can also be explained using a dynamic mechanical model, as the osmotic pressure of the vesicle is passively altered, and when actin coats no longer provide enhanced contractile force, the osmotic pressure passively reaches a mechanical equilibrium with the original contractile force of the actin coat, causing vesicle fusion to pause. Borges et al. studied the release from mouse chromaffin cells using both whole-cell patch amperometry and cell-attached patch amperometry, finding that cells measured with the patch showed nearly double the charge released compared to unpatched cells.^87^ De Toledo et al. likewise used patch amperometry and cell-attached patch amperometry to study the effect of synaptotagmin-7 on the exocytotic release from chromaffin cells, with results indicating that unpatched cells had an average release of 0.62 pC, whereas patch amperometry showed an average release of 3.24 pC in the same cells.^88^ This can be interpreted as the addition of the patch strengthens the actin coat’s force in contracting the Ω-profile, hence forcing more vesicular contents to be released. In contrast, changing the osmotic pressure would also disrupt the existing dynamic equilibrium, altering the vesicle fusion mode. This phenomenon has been reported in several articles. Altering the cell osmotic pressure with hyperosmotic or hypoosmotic solutions would, respectively, promote or inhibit the occurrence of full fusion.^74^^,^^89^ About 20% of the vesicle fusion in hippocampal neurons is kissing and running, but this proportion may increase to over 80% after stimulation with high osmotic solution.^90^ Therefore, constricted fusion can explain various hormonal and neurotransmitter release kinetics changes observed in secretory cells well, implying that vesicle fusion is under biomechanical regulation within a dynamic mechanical equilibrium.

### Microenvironmental stress regulates the fusion release of vesicles in nerve cells

Cells exist in a complex microenvironment, subjected to various types of stress from extracellular matrix, body fluids, and intercellular interactions, maintaining a delicate mechanical balance in the process of vesicle fusion and release. When the body is invaded by disease or external forces (brain tumors, brain edema, traumatic brain injury, concussion, etc.), the biophysical properties of the extracellular environment also change. Cells can sense these stresses and mediate the dynamic mechanical equilibrium of intracellular vesicle fusion and release processes by regulating plasma membrane and underlying actomyosin network properties. The ability of cells to adapt to the constantly changing biomechanical environment is critical for intercellular communication.

#### Tensile stress

Neuronal cells nestled within the protection of the cranium and spinal cord ordinarily remain unexposed to tensile stresses. Nevertheless, external forces from incidents such as vehicular accidents or sports injuries can subject these cells to stretching or compression, leading to nerve damage or concussion. In neurological disorders such as hydrocephalus, brain tumors, and multiple sclerosis, the accumulation of cerebrospinal fluid can cause an increase in intracranial pressure, and the growth of aberrant cells may compress adjacent cells—similarly resulting in tensile stress on neurons. *In vitro* studies have substantiated that tensile stress is an indispensable element in neuronal healing and in the mechanical adaptation of healthy nerves. The negation of tensile stress can have deleterious effects on the nervous system.

##### Vesicle fusion release under tensile stress

Investigations on the neuromuscular junction (NMJ) of Drosophila have revealed that the application of mechanical strains increases the synaptic vesicle (SV) clustering at presynaptic terminals under tensile conditions, while compressive conditions, though reducing the axon tension, do not significantly alter the SV clustering quantity.^16^ On stretching of Drosophila neurons, the axonal tension escalates linearly with the degree of deformation applied and relaxes to a steady-state value over approximately 25 min when deformation remains constant. The steady-state tension in neurons is typically higher than the residual tension. This phenomenon is also exhibited in PC12 neuritic processes and chick sensory neurons, which maintain a rest tension in response to external tensile forces and exhibit a linear relationship between force and length variations.^91^^,^^92^ Actin serves as a structural scaffold at synaptic junctions, clustering vesicles and transporting them to active zones. Concurrently, tensile strain promotes the polymerization of actin and microtubules.^15^^,^^17^^,^^93^ Therefore, the increased axonal tension could facilitate more binding sites for actin-based syanptotagmin-mediated vesicle transport by promoting the aggregation of the actin scaffolds at synaptic regions.^94^^,^^95^ The time required for actin scaffold assembly aligns well with the slow timescale observed when SV clustering is induced by tension.^16^ Moreover, the scaffold does not disassemble immediately upon reduction of tension, resulting in continued SV clustering even after the removal of stretch. This indicates a mechanotransductive capability within neurons, leveraging tensile stress to modulate synaptic structures and thereby vesicle fusion release.

##### Mechanotransductive model for cellular perception of tensile stress

**Figure 3:**
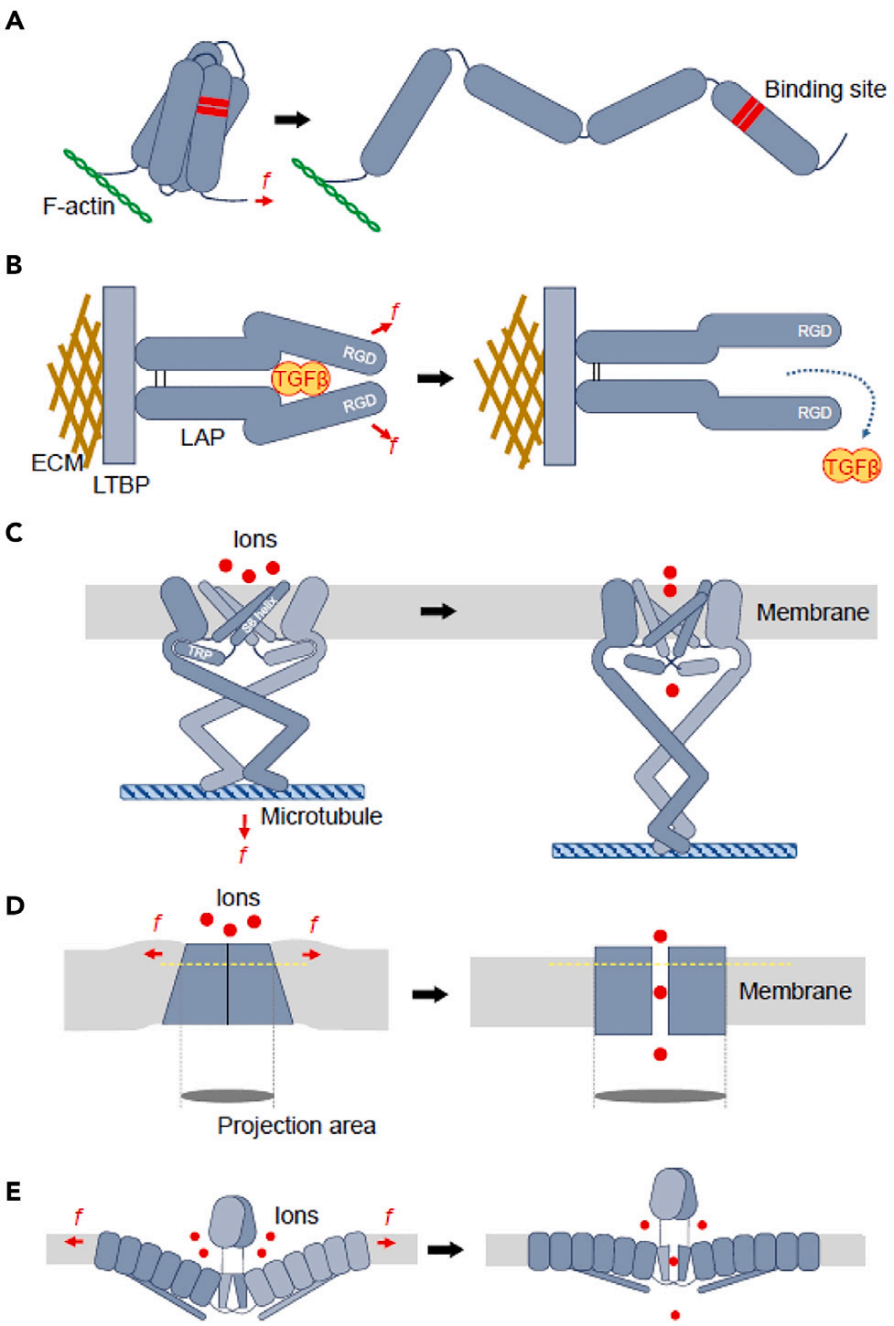
Schematic diagram of mechanical transduction model (A) Adhesive complexes possess the capacity to perceive mechanical loads and experience conformational alterations in response. (B) The latency-associated peptide (LAP) exhibits force-induced changes in its spatial configuration. (C) Mechanical stress is competent in evoking alterations in the tertiary structure of transient receptor potential (TRP) channels, consequently provoking rearrangements in the S6 helix. (D) Applied mechanical forces instigate structural reconfigurations within the wedge-like architecture of the Two-P domain potassium (TRAAK) channel. (E) The mechanical tension within the lipid bilayer is a determinant for inducing conformational transitions in the Piezo1 subunit. Images of (A–E) are reproduced from the study by Lim et al.^96^ with permission from Copyright 2018, BMB Reports.

Cells undergo deformation when exposed to tensile forces, translating this mechanical signal into a biochemical signal. Currently, two principal hypothetical models have been proposed to elucidate this signal transduction process.^96^ The first is known as the “tethering model,” in which proteins anchored at areas of cell-cell or cell-extracellular matrix (ECM) contact are posited as “mechanosensors,” capable of responding to forces and converting them into biochemical signals. When these proteins are mechanically pulled in the opposite direction to their anchored sites, the molecules undergo conformational changes under tension, thereby revealing binding sites for interaction with other proteins (Figure 3A), or disrupting existing protein-protein interactions (Figure 3B), similar to the initiation of signaling cascades involving protein-protein interactions as reported for growth factors or hormones.^97^ The mechanical stretching can also directly induce protein conformational changes, thus modulating related enzyme activities (Figure 3C) and leading to the initiation of cell signaling.^98^

In another “lipid bilayer model,” the cellular lipid bilayer is considered pivotal in sensing mechanical stresses. Forces exerted on the cell can cause deformation of the entire cell, inducing stretching and/or bending in the lipid bilayer within the cell membrane. The transmembrane domains (TMDs) of integral membrane proteins are susceptible to the surrounding lipid bilayer’s conformation.^99^ Hence, the physical property changes in the lipid bilayer induced by mechanical forces can also affect the conformation of the overarching membrane proteins.^100^ Subsequent transition states provoke changes in protein-protein interactions or enzymatic activities (Figures 3D and 3E), serving as the conduit for the transduction of mechanical signals into biochemical ones, a model that has been widely accepted to explain the mechanism of mechanogated ion channel activation.^101^

It is noteworthy that lipid membranes are capable of undergoing a phase transition at temperatures slightly below physiological conditions. The exact position of the phase transition and the corresponding melting temperature are influenced by factors such as lipid composition, pressure, membrane protein characteristics, and pH levels.^102^ At the transition temperature, the membrane exhibits greater permeability, accompanied by ion channel like phenomena even in the complete absence of proteins.^103^ This increased pliability of the membrane implies enhanced facilitation of endocytosis and exocytosis processes. Concomitantly, the propagation of mechanical signals associated with nerve impulses is significantly augmented.^104^

##### Tensile stress modulates vesicle fusion release through ion channels

TREK-1 belongs to the two-pore-domain potassium (K2P) channel family, characterized by a pore domain analogous to other K^+^ channels but distinguished by having two pore-forming domains and four transmembrane helices—two from each subunit—bridged by an extracellular loop. The structural design suggests that TREK-1 channels exist in either a “down” or an “up” conformational state. In the “up” conformation (open state), the TM4 helix shifts upward, opening the ion channel; conversely, in the “down” conformation (closed state), the TM4 shifts downward, preventing ion flux.^105^ Within the lipid bilayer, the “up” conformation presents a cylindrical profile, while the “down” conformation resembles a wedge shape, causing deformation in the lipid bilayer (Figure 3D). The membrane tension induced by mechanical force increases the energetic cost for the wedge-shaped conformation, thereby favoring the cylindrical shape and promoting the mechanical opening of TREK-1 channels under tensile stress (Figure 3D).^106^^,^^107^ Glutamate, the principal excitatory neurotransmitter in the brain, can be directly released via TREK-1 channels, thereby participating in G-protein-coupled receptors (GPCRs) activation,^108^ which are essential for cellular communication governing all critical physiological processes.^109^ When GPCRs are activated, they interact directly with fusion proteins through the G protein Giβγ subunits, subsequently modulating the quantity of neurotransmitter release.^110^

##### Tensile stress regulates vesicle fusion release via membrane tension

**Figure 4:**
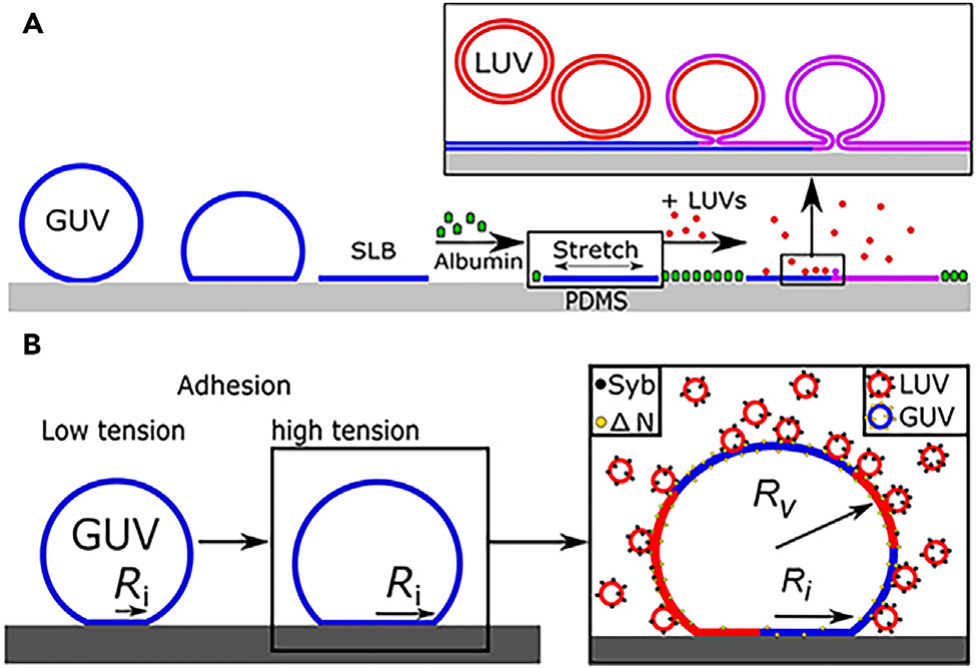
Schematic representation of the two assays manipulating membrane tension and its impact on membrane fusion (A) The observation of tension-dependent fusion of large unilamellar vesicles (LUVs, colored in red) with giant unilamellar vesicles (GUVs, depicted in blue), which have been pre-stretched, is quantitatively assessed. (B) The fusion of LUVs with GUVs adhered to a substrate is accomplished by modulating the membrane tension via altering the degree of adhesion between the vesicles and the functionalized glass surface. Image of (A and B) are reproduced from the study by Kliesch et al.^112^ with permission from Copyright 2017, Scientific Reports.

Tensile stress can also act directly upon membrane tension, increasing it and thereby influencing vesicle fusion release. A growing body of evidence from simulations and experiments demonstrates that heightened membrane tension is essential for successful fusion.^111^^,^^112^^,^^113^ Molecular dynamics simulations have suggested that the tension applied between the vesicle and lipid bilayers enhances their propensity for fusion.^111^ Torben-Tobias Kliesch et al. developed two *in vitro* model strategies to corroborate this concept: one employing expandable polydimethylsiloxane (PDMS) sheets to stretch planar lipid bilayer patches; another manipulating membrane tension through regulation of the adhesion area between giant unilamellar vesicles (GUVs) and the substrate (Figures 4A and 4B).

In the first method, the fusion efficiency marginally increased when the membrane tension reached 3.2 mN m^−1^, with a significant enhancement in fusion rates becoming evident around 4.0–4.4 mN m^−1^. In the second approach, a slight improvement in fusion efficiency occurred at membrane tensions ranging from approximately 0.6–2.6 mN m^−1^, with a notable increase in efficiency around 4.0–5.0 mN m^−1^. In summary, both methods underscored the pivotal role of membrane tension in vesicle fusion efficiency, suggesting a threshold of membrane tension must be overcome to facilitate fusion.^112^

Utilizing atomic force microscopy (AFM) for the force spectroscopy of lipid bilayers has revealed a process in which the AFM tip approaches the surface at a constant velocity and mechanically interacts with the lipid layer at a set tip-to-surface distance. Upon contact, the supported lipid bilayer undergoes elastic compression until the tip penetrates it, resulting in a “jump-to-contact”. This puncturing of the bilayer by the AFM tip is characterized by a horizontal discontinuity in the force-distance curve.^114^ The vertical force at this discontinuity corresponds to the maximum force the bilayer can withstand before rupture, known as the breakthrough force (Fb) or yield threshold force.^115^ This provides a compelling explanation for the necessity of overcoming a membrane tension threshold to facilitate fusion, essentially by overcoming the lipid bilayer’s yield threshold force, thus enabling membrane pore formation.

Lipid bilayers are composed of hydrophilic and hydrophobic surfaces, each playing distinct roles in vesicle fusion. Remarkably, surface adhesion serves a pivotal role in this process, as it governs membrane tension which, in turn, is linked to the spatial distribution of adhesion, thereby coupling vesicle shape changes with adhesion dynamics.^116^ Significant efforts have been made to understand the deformation and rupture of individual vesicles on hydrophilic surfaces. Vesicle shape post-adhesion is determined by an equilibrium between curvature and adhesion energy. The degree of deformation increases with the strength of adhesion. On hydrophilic surfaces, strong adhesion induces bilayer stretching and rupture.^117^ Rupture initiates from pore formation in the highly curved bilayer regions near the substrate,^118^ eventually leading to planar bilayer patches through pore growth.^118^ In contrast, less is known about vesicle adsorption on hydrophobic interfaces. Structural changes involving deformation and rupture on hydrophobic surfaces are more complex. Upon adsorption, lipid carbon-hydrogen tails within the inner leaflet become exposed at the surface, forming a stable contact. Consequently, the deformed vesicle is composed of unbound bilayer and bound monolayer regions. Following rupture, the formation of a monolayer patch necessitates the transition of a bilayer into a monolayer structure.^119^

Tensile stress can also impact membrane tension by acting upon the actin cytoskeleton. When cells are subject to tensile stress, the actin cytoskeleton within distinctly manifests responses to mechanical perturbations.^63^^,^^120^^,^^121^ This in turn affects the membrane tension provided by the actin, impacting the consolidation of Ω-profiles and the release of contents.^58^ Actin cytoskeleton serving a crucial role in regulating vesicle fusion release, there are two primary notions: (1) it functions as a physical/functional barrier during exocytosis, facilitating the introduction of vesicular contents into the plasma membrane;^120^ and (2) it steers vesicles to the fusion site and dictates the dynamics of the fusion pore’s opening and closing, simultaneously propelling the fusion process to its completion.^121^

#### Shear stress

Neurological cells, which are extensively present in both the brain and spinal cord, may experience morphological and functional changes due to shear stress induced by mechanical compression, impact, stretching, or other external forces. Shear stress also plays a role in neural damage or apoptosis in the context of neurological disorders, such as spinal cord injury or Parkinson’s disease. Additionally, as neurons grow and move normally, inevitable interactions with surrounding tissue or other neurons subject them to shear stress.

##### Shear stress modulates vesicle fusion release through activation of ion channels

Shear stress can regulate vesicle fusion release by activating ion channels. The flow transmits forces onto cells through the actin cytoskeleton,^122^ and the TRPV4 channels display increased sensitivity to shear stress following phosphorylation mediated by protein kinase C (PKC) and protein kinase A (PKA).^20^ After the shear stress induced activation of TRPV4 ion channels,^122^^,^^123^ the inflow of extracellular calcium and release from cellular stores triggers vesicle fusion release events.^124^^,^^125^ Integrin linked kinase (ILK) is a serine/threonine kinase, and the GTP enzyme Rac1 in the Rho family can regulate signal transduction and the actin cytoskeleton dynamics.^126^ The inhibition of ILK or Rac1 leads to a considerable reduction in TRPV4 ion channel activation by shear stress, suggesting that shear stress-mediated vesicle fusion release enhancement is regulated through the activation of the ILK/Rac1 pathway.^127^ Another channel Piezo1 has been shown to be activated by shear stress, while cells lacking Piezo1 show a significant decrease in calcium influx in response to shear stress.^128^ This suggests that Piezo1 may also respond to shear stress, indirectly regulating vesicle fusion release by controlling calcium influx.

##### Shear stress regulates vesicle fusion release through activation of GPCRs

**Figure 5:**
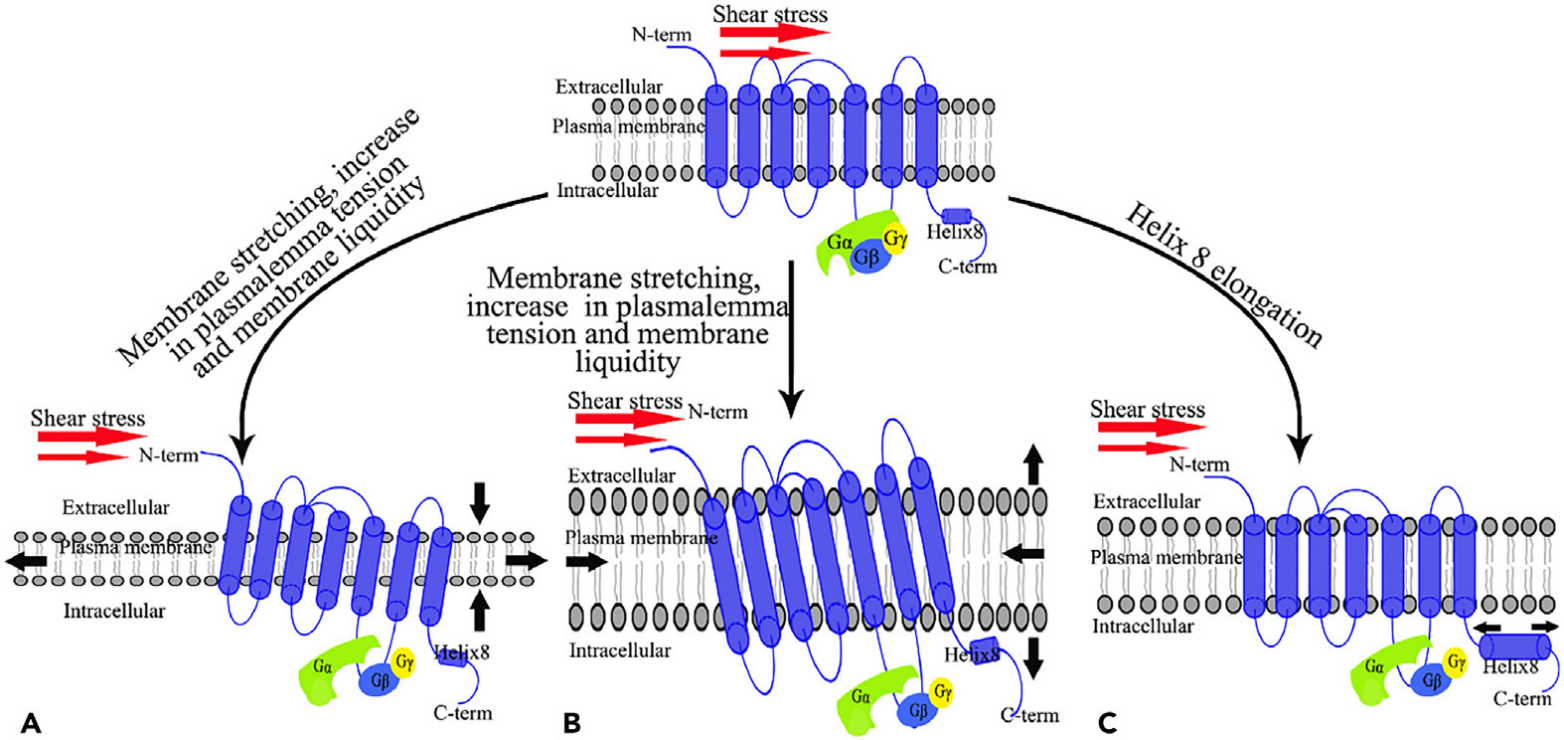
Models of shear stress-activated G-protein-coupled receptor (GPCR) mechanisms (A) Membrane stretching can change the transverse pressure distribution of the plasma membrane; (B) Shear stress causes a non-dependent conformational change in GPCR ligands by increasing membrane tension and fluidity; (C) Shear stress activates G protein through helix 8 extension. Image is reproduced from the study by Hu et al.^155^ with permission from Copyright 2022, Trends in Cardiovascular Medicine.

Stress signals may also activate intracellular signaling pathways directly, thereby inducing vesicle fusion and release events. G protein-coupled receptors (GPCRs) can be activated in response to shear stress, subsequently modulating the initiation of voltage-gated Ca^2+^ channels (VGCCs), which causes Ca^2+^ influx leading to vesicular fusion and release events.^18^^,^^19^^,^^129^ Minor variations in lateral pressure can cause conformational changes in membrane proteins within the phospholipid bilayer; indeed, membrane stretching might adjust the lateral pressure distribution across the bilayer.^130^ Such alterations in the bilayer can prompt GPCRs to assume an active state (refer to Figure 5A). Chachisvilis et al. employed time-resolved fluorescence microscopy and fluorescence resonance energy transfer (FRET) techniques to monitor the conformational dynamics of GPCRs under fluid shear stress. They observed that the conformational changes of GPCRs under such stress reached a saturation point at physiological levels of shear stress (approximately 15 dyn/cm^2^), indicating that shear stress-mediated ligand-independent conformational changes in GPCRs are driven by increased plasma membrane tension and augmented membrane fluidity (as highlighted in Figure 5B). The Helix 8 structure acts as an essential motif through which GPCRs can sense mechanical stress. Under shear stress, Helix 8 elongates, leading to the activation of G proteins, thereby triggering the signal transduction process^131^ (illustrated in Figure 5C).

##### Shear stress may directly promote vesicle membrane fusion

Kogan et al. applied shear stress to vesicles in a sucrose-containing buffer using a Couette flow cell, characterizing the phenomena using dynamic light scattering (DLS) and FRET-based fluorescence measurements, demonstrating that shear stress facilitates membrane fusion events between vesicles. In the shear flow, vesicles responded to the applied stress by developing tension within their membrane and temporarily adopting ellipsoidal shapes.^21^ The highly curved areas of two vesicles, extremities of the ellipsoids, contact with each other, and, due to the high membrane tension, the lipids in apposing bilayers may form a fusion pore.^132^ Membrane curvature stress induced by deformation is known to control vesicle fusion on solid substrates.^133^ When deformation reaches a critical threshold, vesicles rupture and merge, thereby relieving membrane curvature stress. Thus, within cellular environments, people might question whether shear stress could directly stimulate fusion events in the area where vesicles converge with the presynaptic membrane. The presynaptic membrane responds to membrane curvature stress by opening pores, and during the fusion process, vesicles effectively increase the surface area of the presynaptic membrane to counteract the rise in membrane tension caused by shear stress. It is conceivable that shear stress could influence vesicle fusion release through such mechanisms, but this hypothesis requires further experimental confirmation. This knowledge may provide additional insight into therapeutic targets for dysfunctions in areas such as brain lesions, cerebral white matter, and the blood-brain barrier, where shear stress is known to play a significant role.^134^^,^^135^

#### Extracellular matrix stiffness

Previous studies suggest that the stiffness of the extracellular matrix (ECM) can influence the mode of vesicle fusion and release. In pathological conditions, changes in the Young’s modulus of affected tissues, either increase or decrease, correlate with alterations in the mode and frequency of vesicle fusion and release. For instance, in diseases such as Alzheimer’s and Parkinson’s, a reduction in the brain tissue Young’s modulus may lead to a decreased occurrence of the kiss-and-run fusion model^136^^,^^137^^,^^138^; In contrast, diseases such as brain tumors, traumatic brain injury, and multiple sclerosis might see an increased number of vesicle fusion and release events due to an elevated Young’s modulus in brain tissue.^139^^,^^140^^,^^141^

##### Extracellular matrix regulates vesicle fusion and release via integrins

ECM molecules link to the cellular cytoskeleton through integrins. Integrins are heterodimeric receptors composed of alpha and beta subunits.^142^ The activation of integrins by matrix ligands leads to conformational changes that expose intracellular domains capable of binding focal adhesion kinase (FAK) and integrin-linked kinase (ILK) among other focal complex proteins. Integrins cluster cytoplasmic proteins such as vinculin and talin, and actin stress fibers, forming focal adhesions; subsequently, integrin triggers phosphorylation cascades and initiates signaling events.^143^ The cytoskeleton components, actin, and microtubules (MT), play a critical role in vesicle transport and fusion,^82^^,^^121^^,^^144^ and studies have shown that integrins regulate these factors, thereby participating in the control of vesicle fusion and release events.^145^^,^^146^^,^^147^

##### Integrins regulate vesicle fusion release by regulating microtubules

**Figure 6:**
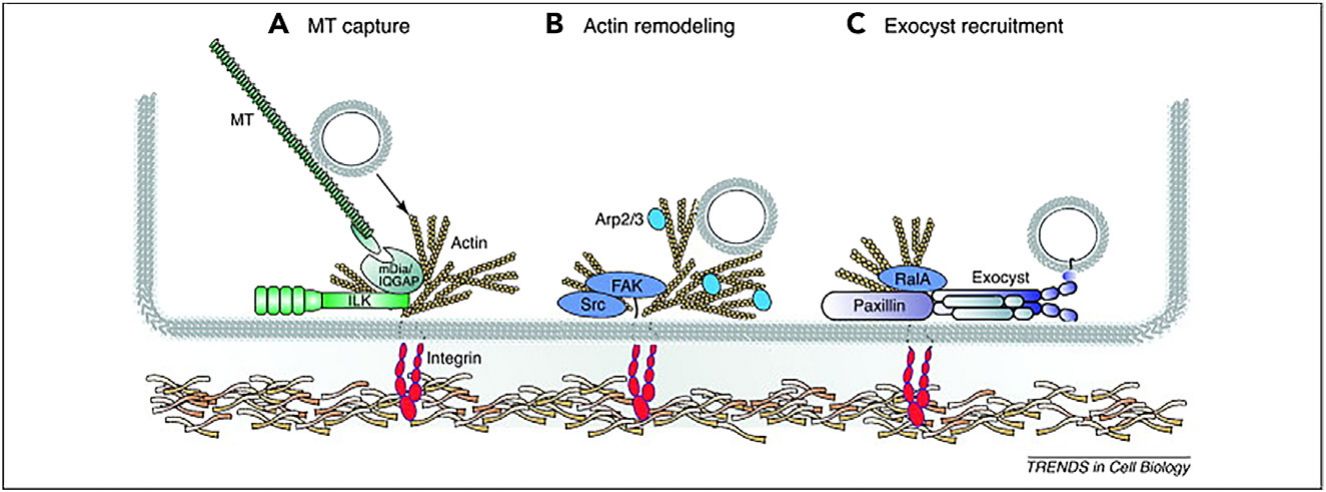
Integrin signaling regulates exocytosis on various levels Integrin signaling has been shown to regulate exocytosis in various cellular systems. The regulation can occur on at least three levels: (A) integrin signaling through the scaffold protein ILK regulates the recruitment of MT-stabilizing proteins to the cell cortex. (B) Integrin regulates actin remodeling through the activity of integrin associated kinases FAK and Src. (C) The newly formed adhesion sites can recruit GTPase RalA, which can promote extracellular vesicle assembly and stimulate downstream fusion activity, leading to the fusion of extracellular vesicle assembly and activated site secretory vesicles. Image is reproduced from the study by Wickstrom et al.^156^ with permission from Copyright 2011, Trends in Cell Biology.

In the preceding sections of this article, the pivotal role of microtubules in the process of vesicle fusion and release has also been delineated. Integrins have been elucidated to modulate microtubule-associated mechanisms, thereby indirectly governing the vesicle fusion and release process. In polarized epithelial cells, MTs are aligned parallel to the apical-basal polarity axis, and the plus ends of MTs are anchored to the plasma membrane via integrin-dependent mechanisms, ensuring local stabilization.^147^^,^^148^^,^^149^ In epithelial cells, integrin receptors α3β1 and α6β4 promote the accumulation of MTs toward the basal cell cortex.^149^ In fibroblasts, integrin receptor α5β1 promotes MT stabilization by activating FAK.^148^ In keratinocytes, the organization and dynamics of MTs are regulated by the β1 integrin effector, ILK, which recruits scaffolding proteins, IQGAP1 and its binding partner mDia, to the cell cortex, thus achieving local capture and stabilization of MTs. In the absence of ILK, vesicles transported near the plasma membrane are unable to fuse (Figure 6).^147^ ILK also regulates α-granule secretion in platelets and VEGF secretion in endothelial cells,^146^^,^^150^ suggesting that ILK may play a role in regulating exocytosis in a broader cellular context. In fibroblasts, integrin adhesion promotes the exocytosis of lipid rafts in an MT-dependent manner,^151^ and even minor changes in the integrin-FAK axis can disrupt microtubule equilibrium, resulting in fewer plasma membrane rafts.^148^

##### Integrins regulate vesicle fusion release by modulating the actin cytoskeleton

The actin cytoskeleton plays a key role in vesicle fusion and release, providing driving force for vesicles and the mechanical foundation for the exocytosis of secretory granules.^121^^,^^144^ Stabilization and de-polymerization of the actin cortex reduce exocytosis, highlighting that the level and quality of cortical actin are crucial for this process.^152^ Studies indicate that exocytosis in neuronal cells depends on local actin remodeling mediated by integrin signaling. Neurons plated on laminin, through a β1 integrin signaling-dependent Rac-Arp2/3 actin remodeling pathway, induce exocytosis.^145^ Notably, this pathway, like MTs, is regulated by FAK. This report, along with the additional finding that nocodazole-mediated inhibition of MT dynamics impairs exocytosis^145^ and neuritogenesis,^153^ strongly suggests that integrins regulate exocytosis by coordinating MT-actin crosstalk (Figure 6).

In summary, integrins act as bidirectional conduits between the cell and its extracellular space, become activated by ECM ligands, then undergo conformational changes. Subsequent exposure of integrin binding sites triggers phosphorylation cascades and initiates signal transduction events. Integrins then regulate exocytosis by coordinating the crosstalk between MTs and the actin cytoskeleton.

### Technical methods for investigating the kinetics of vesicle fusion and release process

Vesicular fusion and release represent the foundational mechanisms for signal transduction in both neuronal and various other cell types. A plethora of essential biomolecules, such as dopamine, epinephrine, norepinephrine, serotonin, and neuropeptides, are secreted into the bloodstream through this pathway, thereby sustaining normal physiological functions. Disorders both genetic and acquired, including cystic fibrosis and neurodegenerative diseases like Alzheimer’s and Parkinson’s, have been linked to aberrations in vesicular fusion and release. Thus, investigating the mechanisms underlying vesicular fusion and release is of paramount importance for understanding and treating relevant conditions. Simultaneously, vesicles have been exploited in targeted drug delivery. Understanding vesicular fusion and release can assist in the development of novel drug transport systems that effectively deliver therapeutics to specific cells by mimicking natural exocytotic pathways. Given that vesicles are nanoscopic particles and their fusion and release occur over microsecond to millisecond timescales, the study of these processes necessitates technologies that offer high sensitivity and high temporal resolution. This paper focuses on the technical methodologies employed in the study of vesicular fusion and release kinetics, elucidating the requirements, benefits, and limitations of various approaches.

### Summary and Prospect

Vesicular fusion and release events maintain a dynamic equilibrium within the cell body under the action of different proteins and structural forces, ensuring the smooth progression of these events. Many related studies have shown that changes in the mechanism of action of the involved proteins or structures can be explained by disturbing this dynamic mechanical balance. At the same time, there is a negative feedback regulation mechanism in vesicular fusion and release; when membrane tension increases and the effect of fusion and release events is enhanced, cells reduce membrane tension through diffusion and endocytosis. Furthermore, through exo-endocytosis processes, local membrane tension gradients are generated, inducing actin polymerization, and promoting other cellular activities.^154^ The feedback loops and cooperation between the vesicle fusion and release events involved in membrane tension and the cytoskeleton can explain the common molecular mechanisms observed in cell dynamics during growth, diffusion, migration, and axon guidance (Table 1).

**Table 1:** Technical methods for investigating the kinetics of vesicle fusion and release process

Technical Method	Technical Principle	Applications	Advantages	Limitations
Atomic Force Microscope(AFM)	The Atomic Force Microscope (AFM) employs a sharp tip attached to a flexible cantilever to probe the cell surface.^157^	1. Measurement of vesicle size.^158^ 2. Observation of the structure and arrangement of SNARE complexes.^159^ 3. Real-time observation of plasma membrane dynamics.^160^	1. Real time observation 2. High resolution 3. Simple sample preparation. 4. Compatibility with optical microscopy techniques. 5. High temporal resolution.^161^	1. Unable to Image the undercut structures. 2. The scanning method has limitations.
Optical Tweezers	Optical tweezers employ optical traps to hold micron-sized polystyrene or silica beads that function as force sensors for manipulating individual macromolecules attached to the beads.^162^	1. Manipulate individual vesicles.^162^ 2. Force film fusion through optical heating.^163^ 3. Manipulate a single SNARE complex.^164^ 4. Real time derivation of protein conformation.^165^	1. High resolution. 2. Manipulate and measure individual molecules. 3. Characterizing thermodynamics and kinetics. 4. Analysis of complex multiscale reaction networks 5. Reversible protein folding and unfolding.	1. Complex structure. 2. Strict environmental requirements. 3. The sample may be damaged by light.
Fluorescence Resonance Energy Transfer (FRET)	Two fluorescent molecules with overlapping excitation spectra can achieve photon transfer from the donor to the acceptor at a distance of 0-10nm.^166^	1. Real-time observation of membrane tension.^167^ 2. Detection of Regulating Molecular Interaction Forces.^168^ 3. Changes in protein conformation and spatial position.^169^	1. Real-time, non-invasive, visible, and *in situ* detection. 2. Generate quantitative data on distance, state, and dynamics.	1. Fluorescence may be quenched. 2. Expensive equipment. 3. Complex data analysis. 4. FP tags might affect protein functionality.
Bio-MicroElectroMechanical Systems (Bio-MEMS)	Integrating multidisciplinary technologies such as electronics, mechanics, and optics into miniaturized systems for micro mechanical structures.	1. Real time monitoring at the cellular scale.^170^ 2. Quantitative measurement of exocytosis.^171^ 3. Simulate biological tissues or extracellular environment.^172^	1. High-precision measurement and operation. 2. Single molecule manipulation. 3. Simulate cellular environment. 4. Real time monitoring.	1. Manufacturing complexity and high cost. 2. Poor stability 3. High material requirements. 4. Complex data analysis.

Although there is significant research on the kinetics of each step of vesicle fusion and release, including vesicle transport, pore formation and closure, and content release, there is a gap in understanding the mechanical changes that connect these stages. Vesicles are transported to the synaptic terminal regions under the dynamic balance of forces from actin, myosin, and microtubules, while the SNARE complexes provide the energy to mediate the opening of fusion pores; the newly formed fusion pores are maintained in a new dynamic balance under the combined action of myosin and membrane tension. How the forces involved in vesicle transport dissipate or transfer to the next step at the end of their transport, and the kinetic factors involved in the initiation of each step, have not been thoroughly reported but are undoubtedly important for answering the mechanisms of vesicle fusion and release.

External stress interference alters the dynamic equilibrium of vesicle fusion and release, causing a corresponding change and constructing a secondary dynamic balance. This paper has analyzed its signal regulation pathways and mechanical transmission methods, but direct observations of mechanical feedback stress changes in the synaptic region and the vesicles themselves under the action of external stress are currently incomplete. Addressing these gaps may provide insights for analyzing treatments for conditions involving similar situations, such as Alzheimer’s disease, Parkinson’s disease, traumatic brain injury (where extracellular matrix stiffness changes), and for cells within brain tumors or abscesses (subject to pulling and compression).

## Limitations of the study

This review has potential limitations. The central thesis of our review is the vesicle fusion and release under dynamic kinetic equilibrium. However, the majority of the research articles selected are based on findings in neuronal cells. Yet, the process of vesicle fusion and release is not limited to neuronal cells alone; it also occurs in secretory cells such as chromaffin cells and pancreatic β cells, which were not included in our study scope. This omission leads to a limitation in the generalizability of the arguments.
